# Comparison of alternative gestational age assessment methods in a low resource setting: a retrospective study

**DOI:** 10.1186/s12884-022-04914-6

**Published:** 2022-07-22

**Authors:** Andrea Pietravalle, Silvia Spolverato, Luca Brasili, Francesco Cavallin, Valentina Gabrielli, Gaetano Azzimonti, Donald Micah Maziku, Dionis Erasto Leluko, Daniele Trevisanuto, Giovanni Putoto

**Affiliations:** 1 Doctors with Africa CUAMM, Padua, Italy; 2 Doctors with Africa CUAMM, Dar es Salaam, Tanzania; 3 Independent statistician, Solagna, Italy; 4 St. John of the Cross, Tosamaganga Council Designated Hospital, Iringa, Tanzania; 5 Department of Woman’s and Child’s Health, University of Padua, Padua, Italy

**Keywords:** Gestational age, Last menstrual period, Ultrasound, Neonatal examination

## Abstract

**Background:**

Accurate gestational age (GA) determination allows correct management of high-risk, complicated or post-date pregnancies and prevention or anticipation of prematurity related complications. Ultrasound measurement in the first trimester is the gold standard for GA determination. In low- and middle-income countries elevated costs, lack of skills and poor maternal access to health service limit the availability of prenatal ultrasonography, making it necessary to use alternative methods. This study compared three methods of GA determination: Last Normal Menstrual Period recall (LNMP), New Ballard Score (NBS) and New Ballard Score corrected for Birth Weight (NBS + BW) with the locally available standard (Ultrasound measurement in the third trimester) in a low-resource setting (Tosamaganga Council Designated Hospital, Iringa, Tanzania).

**Methods:**

All data were retrospectively collected from hospital charts. Comparisons were performed using Bland Altman method.

**Results:**

The analysis included 70 mother-newborn pairs. Median gestational age was 38 weeks (IQR 37–39) according to US. The mean difference between LNMP vs. US was 2.1 weeks (95% agreement limits − 3.5 to 7.7 weeks); NBS vs. US was 0.2 weeks (95% agreement limits − 3.7 to 4.1 weeks); NBS + BW vs. US was 1.2 weeks (95% agreement limits − 1.8 to 4.2 weeks).

**Conclusions:**

In our setting, NBS + BW was the least biased method for GA determination as compared with the locally available standard. However, wide agreement bands suggested low accuracy for all three alternative methods. New evidence in the use of second/third trimester ultrasound suggests concentrating efforts and resources in further validating and implementing the use of late pregnancy biometry for gestational age dating in low and middle-income countries.

## Introduction

Accurate determination of gestational age (GA) is of great importance in clinical practice, allowing correct management of high-risk, complicated or post-date pregnancies and prevention or anticipation of prematurity-related complications [1]. The ultrasound (US) measurement in the first trimester (up to and including 13 6/7 weeks of gestation) is considered the gold standard for GA determination and is followed in accuracy by ultrasound in second and third trimester [1]. In low- and middle-income countries, the availability of prenatal ultrasonography is limited by elevated costs, lack of skills and poor maternal access to health service, making it necessary to use alternative methods [2]. The Last Normal Menstrual Period recall (LNMP) and the neonatal physical and neurological maturity assessments with the New Ballard Score (NBS), constitute reasonable measurements for gestational age when compared to ultrasound, and acceptable methods when assessing gestational age in low-resource settings [3–7]. Compared with first trimester ultrasound, LNMP and NBS have a mean bias of 0.2 and 2.8 days, respectively, with 95% limits of agreement of ± 26 days [2, 8]. LNMP shows high sensitivity (84.7%) and specificity (90.5%) for identifying preterm newborns (< 37 weeks) [2], while NBS has moderate sensitivity (64%) but high specificity (95%) [8]. A modified birthweight-sensitive Ballard method (NBS + BW) seems to improve, in routine clinical practice, the assessment of gestational age and correct for errors caused by low birthweight [9].

This study compared three methods of GA determination (LNMP, NBS and NBS + BW) with the locally available standard (US measurement in the third trimester) in a low-resource setting, under real field conditions. The purpose was to identify the best method for GA determination in a low-resource setting.

## Materials and methods

### Setting

This study was carried out at the St. John of the Cross Hospital of Tosamaganga (Iringa, Tanzania), the only Comprehensive Emergency Obstetric and Newborn Care Center in Iringa Rural District. Designated as referral hospital of Iringa Rural District Council, it serves an estimated population of 265 000 inhabitants, handling approximately 2300 deliveries per year. The hospital has a total of 165 beds, 48 of which are in the maternity department, including 12 obstetrics, 18 in vaginal postpartum and 18 in CS postpartum. A labour room, a neonatal resuscitation room and a Neonatal Special Care Unit are also present [10].

### Patients

All the mother-newborn pairs with complete data on the three different methods of determining GA were included in the study.

### Outcome measures

The agreement in GA estimation between different methods.

### Data collection

All data were retrospectively and anonymously collected from hospital charts and did not contain any information that might be used to identify individual patients. Maternal data included: age, weight, BMI, number of pregnancies, mode of delivery, GA by LNMP recall, GA by ultrasound measurement in the third trimester. Neonatal data included: sex, birth weight, APGAR score, GA by NBS and NBS + BW.

### Definitions

The GA refers to the duration of time between conception and delivery. The LNMP recall is the difference between the first day of the last menstrual period and the delivery date. A US is defined as of the third trimester when executed at 28 0/7 weeks of gestation and beyond [1]. Late ultrasound GA determination was performed using the INTERGROWTH-21st project estimation method [11]. The NBS consists in a procedure, performed postnatally up to 96 h after birth, that asses physical and neuromuscular maturity of the neonate to determine its gestational age [12]. NBS + BW refers to the NBS adjusted considering birth weight in the score calculation [9].

### Comparisons

The US measurement in the third trimester was separately compared with LNMP recall, NBS and NBS + BW.

### Statistical analysis

The sample size calculation was based on information from available literature [8]. Assuming a mean difference of 0 weeks with a standard deviation of 3 weeks, a minimum of 64 subjects were required to have an 80% chance of detecting, as significant at the 5% level, an agreement interval of 8 weeks in the Bland-Altman plot. The final sample size was rounded up to 70 subjects (reaching an estimated power of 85%). Sample size calculation was performed using R 4.1 (R Foundation for Statistical Computing, Vienna, Austria) [13].

Categorical variables were summarized as frequency and percentage. Continuous variables were summarized as mean and standard deviation (SD) or median and interquartile range (IQR). The agreement in GA estimation between different methods was assessed using Bland Altman plot (showing mean difference and 95% agreement limits). The correlation between continuous variables was assessed using Pearson correlation coefficient. Inter-rater reliability between the clinicians was evaluated using intra-class correlation coefficient (ICC) in a subsample of 30 newborns with double assessments. All tests were two-sided and a *p*-value less than 0.05 was considered statistically significant. Statistical analysis was performed using R 4.1 (R Foundation for Statistical Computing, Vienna, Austria) [13].

## Results

The analysis included 70 mother-newborn pairs. Maternal and neonatal characteristics are reported in Table 1. Data on GA by LNMP recall, GA by US, GA by NBS and GA by NBS + BW were available for all newborns (*n* = 70).

**Fig. 1:**
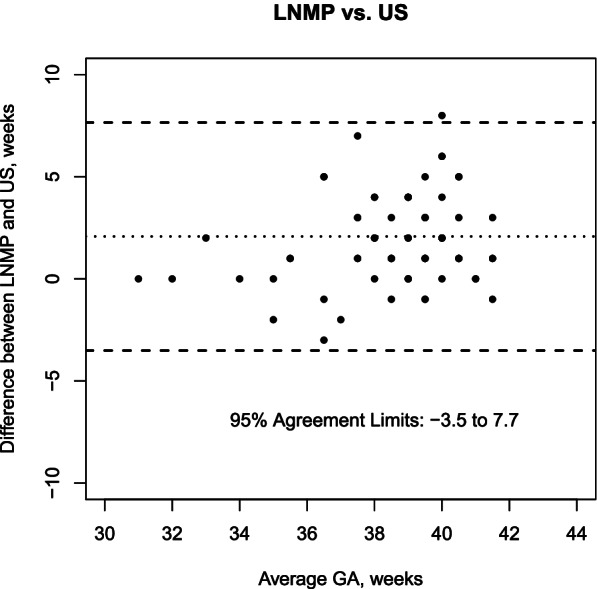
Agreement in GA estimation between LNMP and US: Bland Altman plot (the average of the two measurements for each sample is allocated on the x-axis and the difference between the two measurements on the y-axis)

The agreement in GA estimation between LNMP and US is shown in Fig. 1. Mean difference between LNMP and US was 2.1 weeks (95% agreement limits − 3.5 to 7.7 weeks). There was a mild correlation between difference and average GA value (Pearson correlation coefficient 0.36, *p* = 0.002), which suggested an increasing overestimation of LNMP over US in late GAs. The difference between LNMP and US was not associated with maternal age (Pearson correlation coefficient 0.01, *p* = 0.98), maternal BMI (Pearson correlation coefficient − 0.24, *p* = 0.11) or number of pregnancies (Pearson correlation coefficient 0.01, *p* = 0.93).

**Fig. 2:**
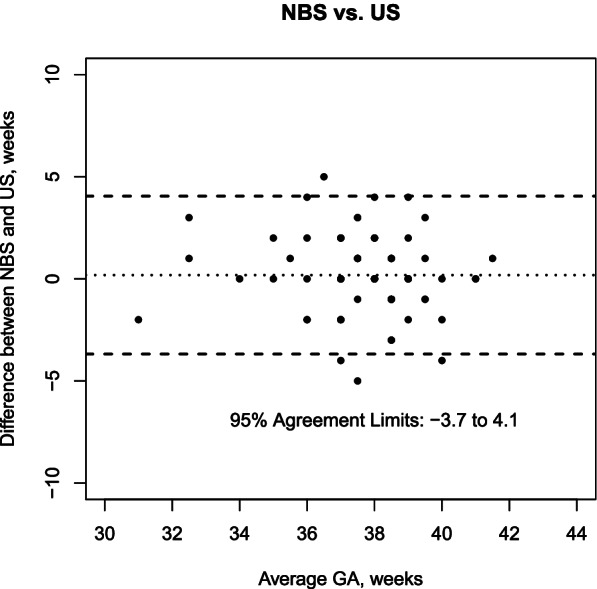
Agreement in GA estimation between NBS and US: Bland Altman plot (the average of the two measurements for each sample is allocated on the x-axis and the difference between the two measurements on the y-axis)

The agreement in GA estimation between NBS and US is shown in Fig. 2. Mean difference between NBS and US was 0.2 weeks (95% agreement limits − 3.7 to 4.1 weeks), without any correlation between difference and average value (Pearson correlation coefficient − 0.11, *p* = 0.38).

**Fig. 3:**
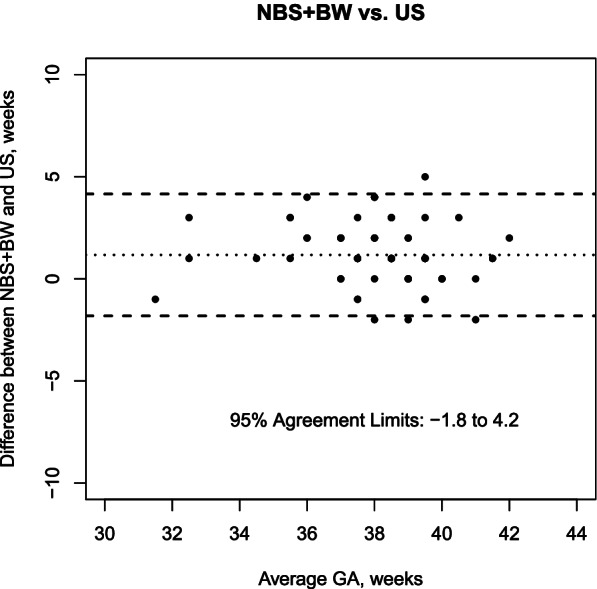
Agreement in GA estimation between NBS + BW and US: Bland Altman plot (the average of the two measurements for each sample is allocated on the x-axis and the difference between the two measurements on the y-axis)

The agreement in GA estimation between NBS + BW and US is shown in Fig. 3. Mean difference between NBS + BW and US was 1.2 weeks (95% agreement limits − 1.8 to 4.2 weeks), without any correlation between difference and average value (Pearson correlation coefficient − 0.15, *p* = 0.20).

In a subsample of 30 newborns, ICC showed good inter-rater reliability for NBS score (ICC = 0.99), NBS neuromuscular subscore (ICC = 0.96) and NBS physical subscore (ICC = 0.95).

**Table 1:** Maternal and neonatal characteristics

Mothers and newborns	Variable	N (%) or median (interquartile range)
Mothers (*n* = 70)	Age, years ^a^	25 (22–28)
	Weight, kg ^b^	64 (59–74)
	BMI, kg/m^2 c^	28.0 (24.2–31.6)
	Number of pregnancies ^a^	3 (1–3)
	Mode of delivery: ^a^ vaginal delivery (assisted) caesarean section vaginal delivery (spontaneous)	1 (1%) 40 (58%) 28 (41%)
Newborns (*n* = 70)	Gestational age according to US, weeks	38 (37–39)
	Males	40 (57%)
	Birth weight, grams	2795 (2702–3345)
	Apgar score at 1 min	8 (7–8)
	Apgar score at 5 min	10 (10–10)

Data not available in ^a^1, ^b^14 and ^c^25 subjects

## Discussion

In our low-income setting, the modified NBS (NBS + BW) was the less biased method for GA determination as compared to NBS alone and LNMP. In addition, the good inter-rater reliability of NBS suggested that it could be consistently used by the health care staff thank to the low subjectivity. On the other hand, our data indicated low agreement between the alternative methods (LMNP, NBS, NBS + BW) and the locally available standard (US measurement in the third trimester).

In low-income countries, the lack of accessible or accurate data on GA is a critical barrier to the correct management of high-risk pregnancies and preterm births. The limited availability of prenatal US suggested the opportunity of investigating alternative methods of GA determination in low-resource settings [2–7]. Unfortunately, we found low accuracy for some alternative methods (LMNP, NBS, NBS + BW) compared with the locally available standard. Overall, our deviations were in broad agreement with previous data reported in available literature [2, 8], thus supporting the unreliability of such alternative methods for GA determination. The deviations of the alternative methods indicated possible underestimation of GA up to 3.7 weeks and overestimation up to 7.7 weeks. As accurate determination of GA is crucial for prevention and anticipation of prematurity-related complications, such magnitude implies the impossibility of discriminating between term and preterm newborns (and among degrees of prematurity) by the health care provider. Given such results, a Reviewer suggested the intriguing idea of combining these methods to improve accuracy of estimated GA. However, NBS and NBS + BW could not be jointly used due to high multicollinearity (NBS + BW values are based on NBS values), hence leading to two options: (i) combining LMNP and NBS, or (ii) combining LMNP and NBS + BW. In both cases, the contribution of LMNP was negligible (data not shown), thus the estimated values were based on NBS or NBS + BW, respectively. Of note, we acknowledge that US measurement in the third trimester may represent a suboptimal reference standard, as dedicated literature suggests US measurement in the first trimester or accurate LMNP recall as the preferred reference standards for testing the validity of alternative methods of GA determination [2, 8]. However, the unavailability of such preferred reference standards forced the use of US measurement in the third trimester as the only viable option in our setting.

Recent evidence suggested that using US in second/third trimester with a novel parsimonious formula might narrow accuracy to ± 10.5 days (between 24 0/7 weeks and 29 6/7 weeks of gestation) and of ± 15.1 days (between 24 0/7 weeks and 29 6/7 weeks of gestation) [14]. These results suggest that concentrating efforts and resources in further validating and implementing the use of late pregnancy biometry for gestational age assessment may be valuable in settings where the preferred reference standards are unavailable.

To our knowledge, this is the first study evaluating the accuracy of the modified birthweight-sensitive Ballard method (NBS + BW) elaborated by Feresu et al. [10], under real field conditions.

Our study has also some limitations that should be considered by the reader. First, US measurement in the third trimester may represent a suboptimal reference standard. Second, the retrospective nature of the study limited data availability. Third, the generalizability of the findings should be limited to similar settings. In addition, our study included few preterm newborns hence caution is suggested in the interpretation of our findings in such subpopulation.

## Conclusions

In a low-income setting, NBS + BW was the least biased method for GA determination as compared with the locally available standard (US measurement in the third trimester). However, wide agreement bands suggested low accuracy for all three alternative methods (LNMP, NBS, NBS + BW. New evidence in the use of second/third trimester ultrasound suggests concentrating efforts and resources in further validating and implementing the use of late pregnancy biometry for gestational age dating in low and middle-income countries.

## Data Availability

The dataset analyzed during the current study is available from the corresponding author on reasonable request.

## Abbreviations

GA: gestational age
US: ultrasound
LNMP: Last Normal Menstrual Period
NBS: New Ballard Score
NBS + BW: NBS + Birth Weight

## Declarations

The study was approved by the Institutional Review Board of Tosamaganga Hospital (protocol number DOIRA/TCDH/VOL.016/5, date 08/09/2021), which waived the need for written informed consent given the retrospective nature of the study and the use of anonymized data from hospital records.
